# Acute Metabolic Decompensation of Isovaleric Acidemia Presenting as Persistent Metabolic Acidosis in a Middle-Aged Man: A Case Report

**DOI:** 10.7759/cureus.67253

**Published:** 2024-08-19

**Authors:** Aparna Ravikumar, Abdelgani Abdelgani, Tadeusz Pawlak, Riya Raphael, Abdelgadir Abdalla

**Affiliations:** 1 Internal Medicine, York and Scarborough Teaching Hospitals NHS Foundation Trust, Scarborough, GBR; 2 Cardiology, York and Scarborough Teaching Hospitals NHS Foundation Trust, Scarborough, GBR; 3 Endocrinology and Diabetes, York and Scarborough Teaching Hospitals NHS Foundation Trust, Scarborough, GBR

**Keywords:** metabolic disease, adult patient, rare case report, metabolic crisis, isovaleric acidemia

## Abstract

Isovaleric acidemia is a rare autosomal recessive inborn error of metabolism that affects the breakdown of the essential amino acid leucine. Acute metabolic decompensation is often triggered by stressors such as surgery, concurrent illness, excessive protein intake, or dehydration. This can lead to a catabolic state with increased endogenous protein turnover, posing a risk of potentially life-threatening crises due to the accumulation of toxic metabolites from incomplete leucine breakdown. Such episodes are rarely observed beyond childhood and adolescence, and the disease’s rarity typically prevents single centers from gaining extensive experience with its full spectrum. This lack of familiarity can be challenging for adult physicians, who may not be well versed in the appropriate management strategies. This case report describes an acute metabolic crisis in a middle-aged man in his late 30s, triggered by an influenza virus infection and presenting as persistent and unresolved metabolic acidosis. We aim to emphasize the importance of early and prompt recognition of metabolic crises in metabolically stable adults with inborn errors of metabolism, particularly for intensivists and acute care physicians.

## Introduction

Isovaleric acidemia is a rare autosomal recessive disorder with a prevalence of approximately 1 in 230,000 [1]. It is caused by mutations in the gene encoding the enzyme isovaleryl-CoA dehydrogenase. This enzyme deficiency disrupts mitochondrial protein metabolism, specifically impairing the breakdown of the essential amino acid leucine [2]. During catabolic states, such as infection, surgery, or febrile illness, increased energy demands lead to the breakdown of endogenous proteins and the release of amino acids, including leucine [3,4]. Excessive protein ingestion can similarly elevate leucine levels. In isovaleryl acidemia, incomplete leucine breakdown results in the accumulation of isovaleryl-CoA and isovaleric acid, as well as metabolites such as isovalerylglycine (IVG), isovaleryl (C5)-carnitine, and 3-hydroxy-isovaleric acid in the blood and urine [5,6]. The accumulation of these metabolites leads to metabolic decompensation, characterized by metabolic acidosis with a raised anion gap due to the presence of these organic acids [6].

Isovaleryl-CoA also inhibits the activity of N-acetylglutamate synthase, which is essential for producing N-acetyl glutamate, a critical activator of the urea cycle enzyme carbamoyl phosphate synthetase I (CPS I) [7]. The deficiency of CPS I impairs the urea cycle, leading to hyperammonemia. Additionally, toxic metabolites can suppress bone marrow function by inhibiting granulocyte production factors, resulting in thrombocytopenia and neutropenia [6,8].

Metabolic decompensation in adults is rarely reported in the literature [5], possibly due to fewer infections, which are significant triggers for metabolic crises [9]. It is also suggested that older individuals might better utilize alternative metabolic pathways to detoxify accumulating metabolites associated with isovaleric acidemia [9].

In the initial evaluation of a patient suspected of a metabolic crisis due to isovaleric acidemia, comprehensive testing is crucial. This includes assessing blood pH and gases to evaluate acid-base status, checking glucose levels to rule out hypoglycemia, and performing a full blood count to identify hematological abnormalities [10]. Renal, liver, and bone profiles should be evaluated to monitor organ function, and amylase and lipase levels should be measured if pancreatitis is suspected. Determining ammonia concentration is essential to identify hyperammonemia, while lactate levels help detect lactic acidosis. Specialist tests, such as plasma or blood spot acylcarnitine, are vital for metabolic profiling. Urine culture and ketone testing are required to detect infections and ketosis, with further investigations like CRP and blood cultures performed as needed to identify inflammation or sepsis [10].

General treatment for isovaleric acidemia involves several key strategies. It is important to avoid triggers of metabolic decompensation, such as fasting, by ensuring adequate carbohydrate intake, either orally or intravenously, and promptly treating fever and other intercurrent illnesses. Patients should adhere to a low-protein diet to minimize leucine intake, avoiding high-protein foods like meat, fish, and dairy [9]. Some patients may follow a more structured low-protein diet, including prescription low-protein food products. L-carnitine supplementation, typically dosed at 50-100 mg/kg/day for adults, helps facilitate the excretion of toxic metabolites [11]. If there is any uncertainty about the patient’s condition, admission for short-term observation is recommended to closely monitor and manage potential complications [9].

## Case presentation

A middle-aged man in his mid-30s presented with a seven-day history of fever, cough, and vomiting. His initial clinical examination was unremarkable. He tested positive for the influenza A virus. Blood tests revealed an elevated CRP level of 26 mg/L (normal range: 0-5 mg/L), and venous blood gas (VBG) analysis showed a raised anion gap metabolic acidosis, as detailed in Table 1. Renal function, electrolytes, ketones, and liver function tests were unremarkable, with the following results: eGFR 71 ml/min/1.73 m² (normal range: 60-89 ml/min/1.73 m²), creatinine 79 µmol/L (normal range: 45-84 µmol/L), urea 7.4 mmol/L (normal range: 2.5-7.8 mmol/L), sodium 138 mmol/L (normal range: 133-145 mmol/L), potassium 5.0 mmol/L (normal range: 3.5-5.3 mmol/L), hemoglobin 143 g/L (normal range: 130-180 g/L), white cell count 3.6 × 10⁹/L (normal range: 3.6-11.0 × 10⁹/L), neutrophils 3.1 × 10⁹/L (normal range: 1.8-7.5 × 10⁹/L), bilirubin 21 µmol/L (normal range: <21 µmol/L), and ketones 0.3 mmol/L (normal range: 0.6-1.5 mmol/L). There was no history of drug intake, diabetes, or renal dysfunction. The patient’s past medical history included Gilbert’s syndrome and isovaleric acidemia.

**Table 1:** VBGs on admission VBG, venous blood gas

VBGs	Values	Reference range
PH	7.29	7.35-7.45
pCO2	3.6 KPa	4.6-6.0 KPa
pO2	6.6 KPa	3.5-5.3 KPa
Bicarbonate	12.8 mmol/L	22-26 mmol/L
Base excess	-11.9 mmol/L	-2 to +2 mmol/L
Lactate	1.4 mmol/L	0.5-2.2 mmol/L

Initial management included fluid resuscitation, oseltamivir, and bicarbonate boluses. VBGs were reassessed after these interventions, as shown in Table 2. However, after 48 hours, the patient exhibited worsening symptoms, including recurrent episodes of coffee-ground vomiting, an inability to retain solids or fluids orally, generalized malaise, and severe lethargy. The VBG continued to show persistent metabolic acidosis, with a negative base excess of -12 and a pH of 7.2, as detailed in Table 3, despite repeated fluid and bicarbonate boluses. Within a few hours, his Glasgow Coma Scale (GCS) score dropped to 8/15, necessitating immediate transfer to the critical care unit.

**Table 2:** VBGs after repeated bicarbonate boluses VBG, venous blood gas

VBGs	Values	Reference range
PH	7.28	7.35-7.45
pCO2	4.3 KPa	4.6-6.0 KPa
pO2	3.4 KPa	3.5-5.3 KPa
Bicarbonate	14.8 mmol/L	22-26 mmol/L
Base excess	-10.7 mmol/L	-2 to +2 mmol/L
Lactate	1.2 mmol/L	0.5-2.2 mmol/L

**Table 3:** VBGs at the time of ICU admission VBG, venous blood gas

VBGs	Values	Reference range
PH	7.25	7.35-7.45
pCO2	4.0 KPa	4.6-6.0 KPa
pO2	3.6 KPa	3.5-5.3 KPa
Bicarbonate	13.1 mmol/L	22-26 mmol/L
Base excess	-12.7 mmol/L	-2 to +2 mmol/L
Lactate	1.3 mmol/L	0.5-2.2 mmol/L

Upon investigating the cause of his acute deterioration, serum ammonia levels were found to be elevated at 88 μmol/L. Further tests revealed significantly high serum isovaleric acid levels at 18.49 μmol/L (normal: <0.5 μmol/L). The patient was diagnosed with acute metabolic decompensation of isovaleric acidemia. The accumulation of toxic metabolites and ammonia led to metabolic encephalopathy. Elevated plasma ammonia is a key feature of metabolic decompensation, requiring prompt attention to prevent life-threatening neurological complications.

Targeted therapy was promptly initiated with the involvement of metabolic specialists, focusing on returning the body to an anabolic state [12]. Correcting acidosis was a major goal due to the disturbed mitochondrial energy metabolism. A complete protein restriction was implemented for the first 48 hours to minimize protein breakdown and further ammonia buildup. An intravenous infusion of 10% dextrose at 2 ml/kg/hour was started to provide high energy levels, preventing further protein breakdown and promoting anabolism. A glucose infusion was accompanied by hourly insulin administration to maintain a strict glycemic range of 7-10 mmol/L. Intravenous carnitine (1 g twice daily) was administered to bind toxic metabolites, enhance their excretion, and conjugate free isovaleric acid into its non-toxic forms, isovalerylcarnitine and IVG, which are excreted by the kidneys.

Within the first few hours of targeted therapy, the patient’s pH returned to 7.4, as shown in Table 4. High-calorie nasogastric feeds were provided for the first 48 hours, and protein was carefully reintroduced while monitoring serum ammonia levels. A comprehensive diet plan was formulated by metabolic nutritionists. Despite initial improvement, the patient developed complications, including bone marrow suppression and pancytopenia (Hb: 100 g/L, WCC: 2.1 × 10⁹/L, Plt: 28 × 10⁹/L, neutrophils: 1.5 × 10⁹/L, lymphocytes: 0.4 × 10⁹/L, monocytes: 0.1 × 10⁹/L, eosinophils: 0, reticulocytes: 15.1 × 10⁹/L, and CRP: 436 mg/L). He required aggressive treatment with broad-spectrum antibiotics for sepsis of unknown origin, which further delayed recovery.

**Table 4:** Blood gases after four hours of targeted therapy

Arterial blood gases	Values	Reference range
PH	7.44	7.35-7.45
pCO2	3.2 KPa	4.6-6.0 KPa
pO2	8.7 KPa	10.0-13.3 KPa
Bicarbonate	16.0 mmol/L	22-26 mmol/L
Base excess	-6.6 mmol/L	-2 to +2 mmol/L
Lactate	2.2 mmol/L	0.5-2.2 mmol/L

Hyperammonemia-induced encephalopathy was resolved with complete protein restriction, eliminating the need for hemofiltration. The patient’s GCS score improved to 12 within four days, and he was transferred to the ward after a full recovery in seven days. He was discharged with long-term L-carnitine supplements to aid in the conjugation and excretion of metabolites and glucose polymer sachets to consume during illness to prevent a catabolic state. This case underscores the critical importance of early recognition and targeted management of acute metabolic decompensation in adults with organic acidemias.

## Discussion

Early identification of metabolic crisis signs and immediate initiation of targeted therapy are crucial to preventing decompensation, reducing mortality, and avoiding neurocognitive sequelae. Understanding the biochemistry of decompensation is essential for planning effective, targeted interventions to reverse metabolic crises. In metabolic disorders with acute decompensation, outcomes are often inversely related to the time elapsed between symptom onset and the initiation of specific emergency treatment [13,14].

Decompensation signs can be subtle, such as lethargy, worsened appetite, or the exacerbation of preexisting neurological symptoms (e.g., movement disorders). These symptoms can be challenging to assess and may present as irritability. It is crucial to listen attentively to patients and their families, as they often notice early changes more quickly than medical professionals [14].

The prompt involvement of a metabolic specialist team is essential for managing these cases. A multidisciplinary approach ensures that the specific needs of patients with metabolic disorders are promptly addressed, leading to improved outcomes [13]. This case highlights the importance of rapid recognition and targeted management of acute metabolic decompensation, even in adults where such events are rare.

## Conclusions

Proactive measures, such as patient education on recognizing early symptoms, prompt dietary adjustments, and rapid medical intervention, are crucial for managing acute metabolic crises in individuals with organic acidemias. These steps not only stabilize the patient’s immediate condition but also contribute to long-term health and the prevention of severe complications. Thus, the early involvement of metabolic specialists and the implementation of a personalized therapeutic plan are essential for the successful management of these rare adult presentations.
